# Polymorphisms of HIV-2 integrase and selection of resistance to raltegravir

**DOI:** 10.1186/1742-4690-7-98

**Published:** 2010-11-29

**Authors:** Danielle Perez Bercoff, Perrine Triqueneaux, Christine Lambert, Aboubacar Alassane Oumar, Anne-Marie Ternes, Sounkalo Dao, Patrick Goubau, Jean-Claude Schmit, Jean Ruelle

**Affiliations:** 1 Laboratoire de Rétrovirologie, CRP-Santé, rue Val Fleuri 84, 1526 Luxembourg, Luxembourg; 2 UCLouvain, AIDS Reference Laboratory, Avenue Hippocrate 54 - UCL5492, 1200 Bruxelles, Belgium; 3 Faculté de Médecine, de Pharmacie et d'Odontostomatologie de Bamako, International center of Excellence Research Mali (ICER-Mali), BP1805 Bamako, Mali

## Abstract

**Background:**

Human Immunodeficiency Virus type 2 is naturally resistant to some antiretroviral drugs, restricting therapeutic options for patients infected with HIV-2. Regimens including integrase inhibitors (INI) seem to be effective, but little data on HIV-2 integrase (IN) polymorphisms and resistance pathways are available.

**Materials and methods:**

The *integrase *coding sequence from 45 HIV-2-infected, INI-naïve, patients was sequenced and aligned against the ROD (group A) or EHO (group B) reference strains and polymorphic or conserved positions were analyzed.

To select for raltegravir (RAL)-resistant variants *in vitro*, the ROD strain was cultured under increasing sub-optimal RAL concentrations for successive rounds. The phenotype of the selected variants was assessed using an MTT assay.

**Results:**

We describe *integrase *gene polymorphisms in HIV-2 clinical isolates from 45 patients. Sixty-seven percent of the integrase residues were conserved. The HHCC Zinc coordination motif, the catalytic triad DDE motif, and AA involved in IN-DNA binding and correct positioning were highly conserved and unchanged with respect to HIV-1 whereas the connecting residues of the N-terminal domain, the dimer interface and C-terminal LEDGF binding domain were highly conserved but differed from HIV-1. The N155 H INI resistance-associated mutation (RAM) was detected in the virus population from one ARV-treated, INI-naïve patient, and the 72I and 201I polymorphisms were detected in samples from 36 and 38 patients respectively. No other known INI RAM was detected.

Under RAL selective pressure *in vitro*, a ROD variant carrying the Q91R+I175M mutations was selected. The Q91R and I175M mutations emerged simultaneously and conferred phenotypic resistance (13-fold increase in IC_50_). The Q91R+I175M combination was absent from all clinical isolates. Three-dimensional modeling indicated that residue 91 lies on the enzyme surface, at the entry of a pocket containing the DDE catalytic triad and that adding a positive charge (Gln to Arg) might compromise IN-RAL affinity.

**Conclusions:**

HIV-2 polymorphisms from 45 INI-naïve patients are described. Conserved regions as well as frequencies of HIV-2 IN polymorphisms were comparable to HIV-1. Two new mutations (Q91R and I175M) that conferred high resistance to RAL were selected *in vitro*, which might affect therapeutic outcome.

## Background

Patients infected with human immunodeficiency virus type 2 [1] generally progress slowly towards immunodeficiency [2], and the majority are not eligible for antiretroviral (ARV) therapy. The therapeutic arsenal developed against HIV-1, however, is reduced for HIV-2-infected patients as HIV-2 is naturally resistant to all available non-nucleoside reverse transcriptase inhibitors (NNRTI) and to the fusion inhibitor enfuvirtide [3-7]. Moreover, HIV-2 has reduced sensitivity to some protease inhibitors (PI) [6-9] and a lower genetic barrier to resistance to other PIs compared to HIV-1 [10,11], leading to more rapid virologic failure [12]. Recent drug classes such as integrase inhibitors (INI), and more specifically the strand transfer inhibitors (INSTIs) raltegravir (RAL) and elvitegravir (EVG), represent promising treatment options for HIV-2. *In vitro*, phenotypic susceptibility of clinical HIV-2 strains was comparable to that of HIV-1 [13,14].

As with other ARV classes, INI escape mutants may emerge under suboptimal drug concentrations. In HIV-1-infected patients failing an INI-containing regimen, three distinct resistance pathways involving Y143R, Q148H/R/K or N155 H have been described. The Q148 H mutation in combination with the G140 S secondary mutation confers the highest level of resistance to RAL (> 1000-fold) together with the highest replicative capacity *in vitro *[15,16]. RAL resistance is not well documented for HIV-2, although cases of therapy failure have been associated with the emergence of variants carrying the Y143C, Q148K/R, or N155 H mutations, including Y143Y+T97A or Q148K, or Q148R+G140 S [1,17-19]. The N155 H substitution in conjunction with secondary mutations conferred HIV-2 strains a 37-fold increase in RAL IC_50 _[18], suggesting that HIV-2 can embrace the N155 H resistance pathway, although recent data suggest that this mutational pathway might be favored in the IN context of group B strains [1].

The IN proteins of both viruses share the same structure. Despite only 40% identity at the nucleotide level, HIV-1 and HIV-2 share 65% similarity at the amino acid level.

IN catalyzes integration of the provirus into the host cellular DNA. IN is derived from the Gag-Pol polyprotein precursor, and IN dimers join to form a homotetramer. Each monomer consists of three different domains. The N-terminal domain (NTD, AA 1-49) consists of 4 α-helices arranged as a three-helix bundle stabilized by a Zinc atom binding to H12, H16, C40 and C43. The NTD is involved in IN dimerization: more specifically, the N-terminal tail and the first half of helix α3 mediate dimer interface through hydrophobic AA F1, L2, I5, P29, L31, V32 and hydrophilic Q35 [20-22]. The catalytic core domain (CCD, AA 50-212) contains the conserved catalytic triad D64, D116, E152 (DDE motif). These three residues form a pocket binding an Mg-bivalent cation. The flexible loop encompassing residues F139 to G146 and the amphipathic α-helix spanning residues S147 to V165 of the CCD ensure direct binding to DNA and correct positioning of viral DNA to the IN catalytic residues. The C-terminal domain (CTD, AA 213-288) is composed of six α-helices and two anti-parallel β-sheets and is the least conserved in HIV-1. The CTD contains sequences involved in multimerization, a non-specific DNA recognition domain as well as a nuclear localization signal (NLS). The CTD is also thought to interact with reverse transcriptase (RT) [23].

Despite numerous studies investigating the diversity of HIV-1 IN, little is still known about the HIV-2 IN, and most studies involved limited patient numbers. Here we further investigate the conserved and polymorphic positions of the *IN *gene in clinical samples from HIV-2-infected patients. In addition, we report novel resistance mutations selected under RAL pressure *in vitro*. The genotypic and phenotypic characteristics of the HIV-2 IN reported here should contribute to building and fine-tune HIV-2 specific algorithms for the genotypic interpretation of resistance to INIs.

## Results and Discussion

### Clinical samples

**Figure 1:**
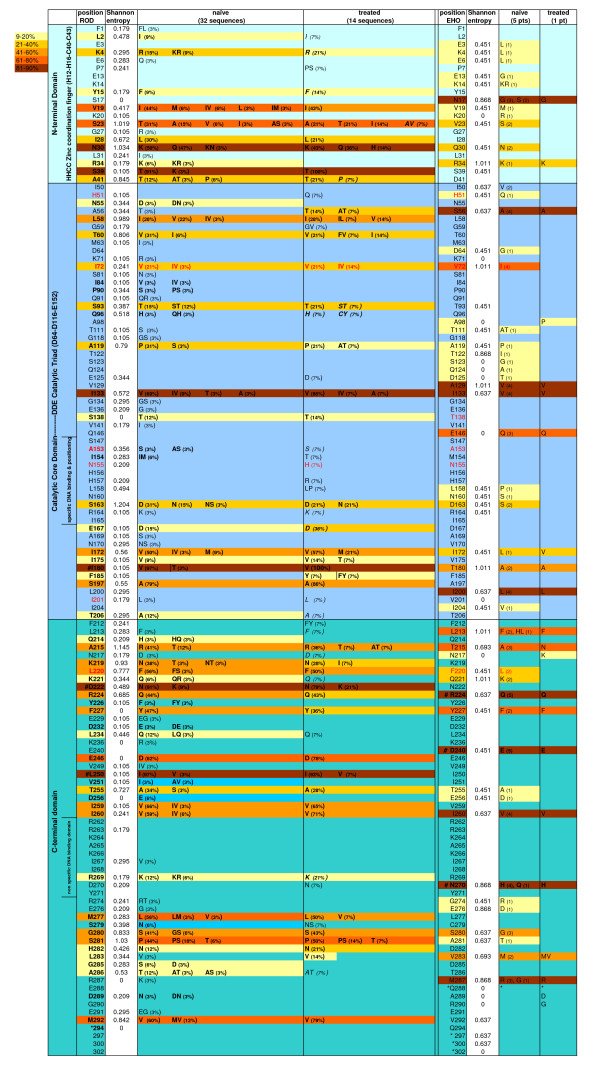
**HIV-2 group A and group B IN polymorphisms**. Polymorphisms of the HIV-2 group A IN sequences from 32 treatment-naïve and 14 treatment-experienced patients, and HIV-2 group B IN sequences from 5 treatment-naïve and 1 treatment-experienced patients are reported with respect to the ROD and EHO reference sequences respectively. Stop codons are marked with a star (*). Positions that were always polymorphic are marked with a hash (#). Positions known to confer resistance to INIs in HIV-1 or HIV-2 are indicated in red in the reference sequence; polymorphisms detected in patient sequences that are known to be associated with resistance to INIs are indicated in red, whereas polymorphisms of unknown impact at those positions are in black. For group A sequences, the frequency (percentage) of each of the polymorphisms is indicated in brackets. For group B sequences, the number of patients in which the polymorphism was detected is indicated in brackets. Only positions where variations were detected are reported. Positions mutated at least twice are highlighted in bold, except when both mutations were detected in longitudinal samples from the same patient. When all the polymorphisms in sequences from treatment-experienced patients were already present in the corresponding baseline samples, they are marked in *italics*; if polymorphisms in the treatment-experienced sequences are redundant with the corresponding baseline sample, they are not highlighted and counted as polymorphisms.

Fifty-two IN sequences derived from 45 patients were analyzed: 46 sequences were from patients infected with HIV-2 group A strains (32 sequences were from treatment-naïve patients and 14 from treatment-experienced patients), and 6 from patients infected with group B strains (5 treatment-naïve patients and 1 treatment-experienced patient). All the ARV-treated patients were INI-naïve. The main epidemiological, immunological and clinical data of the 45 patients included in this study are summarized in Table 1. We report IN polymorphisms for both groups with respect to the reference sequences ROD (group A) and EHO (group B) (Figure 1), but further IN polymorphism analyses are restrained to group A sequences only, because only 6 group B strains were available.

**Table 1:** Patient epidemiological and clinical data.

		Number of patients	Percentage
HIV-2 group	A	39	86.67
	B	6	13.33

Gender	Female	24	53.33
	Male	21	46.67

Transmission	Heterosexual	36	82.22
	Homo-bisexual	3	6.67
	Transfusion	2	4.44
	MTCT	2	2.22
	IVDU	1	2.22
	unknown	1	2.22

Country of origin	Europe	10	22.22
	*Portugal*	*4*	
	*Belgium*	*4*	
	*France*	*1*	
	*Germany*	*1*	
	Sub-Saharan Africa	34	75.56
	*Mali*	*17*	
	*CapeVerde*	*4*	
	*Ivory Coast*	*4*	
	*Guinea*	*2*	
	*Guinea Bissau*	*2*	
	*DRCongo*	*2*	
	*Burkina Faso*	*1*	
	*Sub-Saharan unknown*	*2*	
	Other: Nepal	1	2.22

CDC Stage	A	23	51.11
	B	9	20
	C	13	28.89

ARV therapy	Naive	30	66.67
	Treated (INI naive)	15	33.33
	*NRTIs only*	*6*	
	*NRTI + PI*	*8*	
	*unknown*	*1*	

Age (years, median)		42 [12-78]	

CD4 counts	Naïve	(cell/mm^3^)	
	*Mean, SD*	520 (± 303)	
	*Median, range*	454 [30-1080]	
	Treated (INI naïve)		
	*Mean, SD*	331 (± 283)	
	*Median, range*	286 [6-950]	

Plasma viral load	Naïve	(copies/ml)	
	*Mean, SD*	36,304 (± 74,665)	
	*Median, range*	5420 [Und-351,000]	
	Treated (INI naïve)		
	*Mean, SD*	94,293 (± 188,249)	
	*Median, range*	11,350 [Und-540,000]	

(INI: integrase inhibitors; IVDU: intra-venous drug user; MTCT: mother to child transmission; SD: standard deviation; Und: undetectable viral load)

#### IN Variability and polymorphisms

HIV-2 IN length differed from HIV-1 and between groups A and B: group A IN was 293 AA long, and 4 sequences harbored a second in-frame stop codon at position 297, whereas group B sequences carried stop codons at positions 288, 297, 300 or 302. Viral strains from 4/6 patients carried only one stop codon, one patient's viral strains carried 2 stop codons and one patient's viruses carried 3 stop codons. It is unclear why the HIV-2 IN is longer than the HIV-1 IN, and whether such differences in the length of the IN protein of group B strains play a direct role in enzyme activity. It is conceivable that, because the C-terminus of IN is involved in host DNA binding and positioning, its length might contribute to stabilizing the enzyme onto the substrate. Clonal and biochemical studies would be needed to clarify this issue.

Overall, 97/293 (33.1%) positions in group A IN supported AA changes with respect to the ROD reference sequence, and a total of 131 mutations were identified. This variability is comparable to that reported for the HIV-1 IN (67% of the residues are conserved) [15]. Because of the limited sample size, true polymorphisms are difficult to distinguish from isolated variants. We therefore report positions tolerating a change and positions at which mutations were detected at least twice. Of the 97 variable positions, 61 were mutated at least twice (Figure 1 and Table 2), in line with figures previously reported by Roquebert *et al*. [14]. Fifty-five of the 131 variations resulted from conservative mutations: 24 V<->I < - > L substitutions at 20 positions, 20 A<->S < - > T mutations at 16 AA positions, 7 K < - > R mutations and 5 D < - > E (Figure 1).

**Table 2:** IN variability and polymorphism frequencies in treatment-naïve and treatment-experienced HIV-2 infected patients

	Gene	mutated positions	p	Positions with ≥ 2 mutations	p
**All patients**	**IN**	97/293 (33.1%)		61/293 (19.8%)	
	**PR**	46/101 (45.5%)	**0.030**	33/101 (32.6%)	**0.022**
	**RT**	173/439 (39.4%)	0.086	118/439 (26.8%)	0.080

**Treatment-naïve**	**IN**	94/293 (32.1%)		58/293 (19.8%)	
	**PR**	37/101 (36.6%)	0.323	21/101(20.8%)	0.885
	**RT**	155/439 (35.3%)	0.202	96/439 (20.9%)	0.518

**Treatment-experienced**	**IN**	63/293 (21.5%)		42/293 (14.3%)	
	**PR**	35/101 (34.6%)	**0.006**	23/101(22.7%)	0.061
	**RT**	112/439 (25.5%)	0.13	65/439 (14.8%)	0.915

Variable and polymorphic position frequencies in IN with respect to RT and to PR were compared in treatment-naïve and treatment-experienced patients. Variable positions and positions supporting variability at least twice are reported. Polymorphism frequency was compared using a Fisher exact test, and considered statistically different when p < 0.05.

Overall variability within IN was higher in treatment-naïve (32.1%) than in treatment-experienced patients (21.5%), and 58/293 (19.8%) and 42/293 (14.3%) positions featured at least 2 AA changes in treatment-naïve and in treatment-experienced patients respectively (Figure 1 and Table 2). When compared to the rest of *pol*, IN was as variable as RT (p > 0.05), and less variable than PR (p = 0.03) even when the comparison was restricted to positions that varied at least twice (p = 0.022) (Table 2). When further sub-divided according to treatment experience, viruses from treatment-naïve patients featured similar variability in all 3 genes, while viruses from treatment-experienced patients featured similar variability in IN and RT and slightly higher variability in PR (Table 2), even when only polymorphisms occurring at least twice were considered: 22.7% in PR against 14.3% in IN and 14.8% in RT (Table 2), although this difference did not reach statistical significance, probably owing to the small sample size (14 patients). When variability of IN and PR in treated patients was restricted to the 7 patients infected with group A strains that had received a PI-based regimen, polymorphism frequency in PR was similar to IN and RT: 29/101 (29%) of mutated positions, and 14/101 (14%) positions were mutated at least twice.

Some AA (residues L2, R34, N55, I84, P90, A153, M154, T206, Q214, K221, Y226, D232, V251, D256, S279, G285, A286, D289) were found to support variability at least twice in sequences from treatment-naïve patients, but not in sequences from treatment-experienced patients, probably reflecting the selection of particular IN strains or the counter-selection of certain polymorphisms under NRTI-selective pressure. Indeed, IN and RT are thought to interact [23], and NRTI-selective pressure might therefore also constrain IN. This observation contrasts with one very recent comparative study reporting increased diversity of HIV-1 RT and IN under RTI selective pressure, particularly at IN positions that are thought to interact with RT, such as M154, G163, V165, T206 [24]. Because the HIV-2 IN naturally harbors the AA that were mutated in HIV-1 IN under RTI pressure (V165 in HIV-1 is I165 in HIV-2, M154L in HIV-1 is I154 in HIV-2), it is possible that the HIV-2 IN sequence is naturally more prone to support the changes in RT induced by the emergence of RTI resistance mutations.

#### Analysis of IN polymorphisms

We further investigated variability within each sub-domain of HIV-2 IN. The CCD was the most conserved and the CTD the most variable, in line with previous reports [14]. As for the IN gene as a whole, variability and polymorphism frequencies decreased with treatment experience within each subdomain: for treatment-naïve patients, 15/49 positions were variable within the NTD (of which 10/49 were mutated at least twice), 41/162 (of which 20/162 were mutated at least twice) within the CCD, and 39/81 (of which 28/81 were mutated at least twice) within the CTD; for treatment-experienced patients, 10/49 positions supported variability (8/49 mutated at least twice) within the NTD, 24/162 (16/162 mutated at least twice) within the CCD, 25/81 (18/81 mutated at least twice) within the CTD (Figure 1).

The HHCC Zinc coordination motif, the DDE catalytic triad, and the RKK motif were fully conserved and unchanged with respect to HIV-1 (Figure 1) [14,25-27]. Residues involved in dimer-dimer interaction (NTD polar residues K/R14, N18 and Q44 and CCD residues K160, Q168 and K186) or in multimerization of the enzyme (connecting residues 47-55 and side chains R20 and K34 which interact with CCD side chains T206, Q209 and E212 through hydrophilic contacts in HIV-1 [28]) were all highly or fully conserved (Figure 1) [28,29]. Residues ensuring DNA binding and correct positioning of viral DNA to the IN catalytic residues were also highly conserved. These include the DNA binding residues of the CCD flexible loop (AA F139 to G146) and amphipathic

-helix (AA S147 to V165 in HIV-1, S147 and I165 in HIV-2) involved in direct binding and correct positioning of viral DNA to the IN catalytic residues, the strip of positively charged residues extending from the CCD and the RKK motif (R231, K258, K266), as well as charged residues Q148, E152, N155 and K159 that contact negatively charged viral DNA molecules (Figure 1). Finally, residues 150 to 196 of the CCD, containing a positively charged stretch extending from the CCD through the CTD and that interact with the HHCC Zinc coordination motif of the adjacent monomer, and the C-terminal LEDGF/IN binding domain involving L102, T125, A129, W132, Q168, E170, H174 and M178, were also all highly conserved in HIV-2. AA 34, a Lys in HIV-1, is involved in PIC binding; Arg was found at position 34 in the majority (42/45) of HIV-2 strains, and supported variability to the conserved R34K in 3 sequences. In contrast, CTD residues 195-225 within the

-helix, which are involved in binding to the CCD, featured surprisingly high variability (11/30 positions with at least one AA variation), particularly considering that their interacting counterpart (the CCD highly conserved residues 150-196) tolerated low variability (Figure 1). The main polymorphisms and polymorphism distribution detected in our cohorts did not differ much from those previously reported by Roquebert *et al*. for HIV-2 group A strains [28]. The low variability tolerated at positions involved in enzyme multimerization, catalytic activity and DNA positioning and binding confirm the crucial role of these AA in IN efficacy and viral replication.

The Q96 H mutation has been described to increase infectivity in HIV-1 and in HIV-2 by improving specific interactions with other viral components comprising the initiation complex and thereby increasing the initiation of reverse transcription [30]. Q96 H was detected in sequences from 2 patients for which longitudinal samples were available: one patient maintained the Q96 H substitution after NRTI+PI-based therapy whereas the other patient evolved to a Q96C/Y mixture. Further experiments to assess the replicative capacity of these viruses would be required to assess the impact of Q96H/C/Y substitutions in the genetic context of HIV-2. Other polymorphisms previously described to favor the initiation of reverse transcription in HIV-2, such as the K127E and/or the V204I substitutions within IN, or to increase viral fitness, such as the RT V197I mutation [30] were not detected in sequences from these patients, nor from any other patient.

3 positions were fully polymorphic with respect to the ROD reference (I180V/T, and D222N/K and L250I/V) independently of treatment experience, as previously described [14]; and 3 positions were highly polymorphic: N30K/Q (100% of treatment-naïve samples and 93% of the treatment-experienced samples), S39T/A (94% of the treatment-naïve samples and 100% of the treatment-experienced samples) and I133V/A/T (84% of the treatment-naïve samples and 100% of the treatment-experienced samples) (Figure 1). The impact of these polymorphisms remains to be determined.

#### INI-associated resistance mutations

No mutation described to be associated with INI-resistance in HIV-1 or HIV-2 was detected in our cohort, except for a N155 H substitution in the viral sequence from one treatment-experienced, INI-naïve patient (Figure 1). Other resistance-associated mutations present in that sample included RT mutations M184V and S215Y, and PR mutations V33I, I50V, I54M and I89V. At sampling time, in 2007, RAL was not commercially available, and HIV-2 patients were not included in RAL phase II-III clinical trials, arguing against the hypothesis that the N155 H mutation emerged as a resistance mutation *per se *or that an INI-resistant strain was transmitted. These facts are rather suggestive that the mutation was present as a polymorphism within the patient's variants in which other mutations within RT or PR emerged, or were selected under NRTI+PI selective pressure. Polymorphisms M154T and H157R at neighboring positions were detected only in the sample from this patient, and their impact is unknown. The presence of major RAL and EVG resistance associated mutations prior to therapy including INIs has not been reported for HIV-2 IN to our knowledge. Presumably the presence of this mutation will compromise INI-based regimens, as the N155 H substitution has been associated with RAL-based therapy failure in HIV-2 [1,17-19].

Ile was generally found at positions 72 and 201 in HIV-2 group A IN. I72 was present in 32 patients infected with group A strains and 4 patients infected with group B strains, while I201 was found in all but one group A strains, but was absent from all group B viruses. Mutations V72I and V201I have been described to be selected in HIV-1 IN under EVG selective pressure *in vitro *[26], but their weight on compromising the response to EVG of patients infected with HIV-2 remains to be ascertained. Conservative polymorphisms S138T and A153 S were detected in 12% and 6% of all sequences respectively, but mutations S138A/K and A153Y, associated with decreased susceptibility to RAL and/or to EVG [31], were never detected. Taken together, these data confirm previously reported polymorphisms in HIV-2 [14] and may provide a molecular basis to the positive outcome of RAL use in the clinic [13,15-17], as the polymorphisms detected at positions 72, 138, 153 and 201 do not seem to decrease basal susceptibility to RAL [14], and may be excluded from HIV-2 RAL specific genotypic prediction tools. Their potential impact on susceptibility to EVG and to other investigational INIs is difficult to predict with certainty and would warrant further investigation.

#### Selection of RAL resistance associated mutations in vitro

In order to gain further knowledge on the emergence of resistance to RAL in the context of HIV-2, the ROD strain was cultured *in vitro *in the presence of increasing sub-optimal RAL concentrations starting from 0.001 nM (ROD RAL IC_50 _was 0.00395 nM). After successive increases of RAL concentration, a variant carrying the Q91R and I175M mutations emerged in the population. This variant outgrew an earlier transient variant harboring the I84I/R + L99L/I substitutions when cultured in the presence of 0.027 nM RAL, as well as the parental wild-type ROD strain when RAL concentration was increased to 0.082 nM.

**Figure 2:**
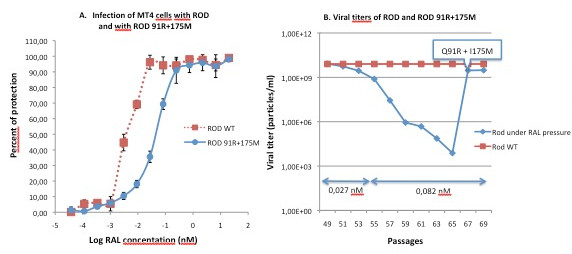
**Phenotypic impact of mutations Q91R and I175M on susceptibility to RAL (A) and on viral titers in vitro (B)**. (A) 3 × 10^4 ^MT-4 cells were infected with 3 × 10^6 ^TCID_50 _(M.O.I. of 100) of ROD or ROD-Q91R + I175M variant in the presence of serial RAL dilutions ranging from 3.763 × 10^-5 ^nM to 20 nM. Infection was quantified by measuring MTT in culture supernatants after 3 days. Infections were performed in quadruplicate wells. 4 independent experiments were performed. The percentage of protection (PP = (O.D. measured - O.D. infected cells without RAL)/O.D. uninfected cells - O.D. infected cells without RAL) × 100). RAL IC_50 _corresponds to the RAL concentration that protects 50% of the culture from virus-induced cytopathic effect, i.e. inhibition of infection. Percent protection is reported as a function of log_10 _RAL concentration (nM). The mean of 4 independent experiments, each performed in quadruplicate wells, is presented. (B) Two million MT-4 cells were infected with 2 × 10^8 ^TCID_50 _of HIV-2 ROD for at least two hours, then washed and cultured in the presence of RAL. New infections were performed twice a week, and virus titers were determined once a week by RT-PCR. Suboptimal concentrations were used during the first passages, then raised gradually by 3-fold. The titers are only shown for the two last drug increases, when the variant carrying Q91R and I175M mutations was selected.

The phenotypic impact of the Q91R and I175M mutations on susceptibility to RAL was assessed *in vitro *using an MTT assay as described previously [32]. As shown in Figure 2, the Q91R and I175M substitutions conferred substantial phenotypic resistance to RAL *in vitro *with a 13.2-fold increase in mean IC_50 _(Mean IC_50 _ROD: 0.00395 nM, IC_50 _range: 0.0036-0.0043 nM; Mean IC_50 _ROD-Q91R + I175M: 0.0424 nM, IC_50 _range: 0.0358-0.054 nM). Although the Q91R substitution has been described previously as a IN polymorphism [19], this is the first time, to our knowledge, that these mutations are associated with resistance to RAL. Viral replicative capacity did not seem to be affected by the Q91R and I175M mutations as the double mutant virus reached the same viral titer as the wild type virus in the absence of RAL pressure.

To assess the potential clinical impact of these mutations, we searched whether they emerged naturally among the IN sequences of our HIV-2 cohort. The Q91R mutation was detected in one isolate from one treatment-naïve patient, and AA I175 was wild-type in that sample. I175V and I175T polymorphisms were detected in samples from 4 patients and 1 patient respectively, but the I175M mutation was never detected (Figure 1).

**Figure 3:**
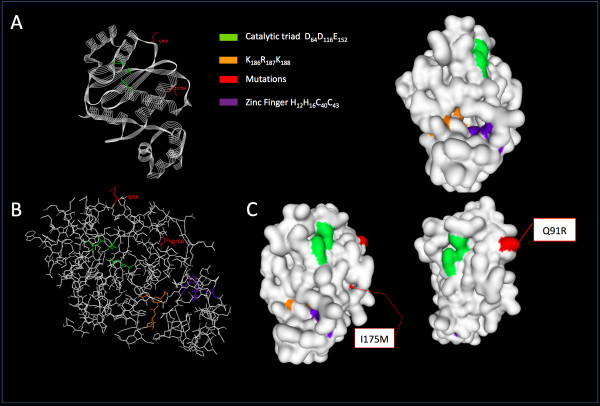
**Positions selected *in vitro *under RAL pressure in an HIV-2 integrase 3D model**. A 3D-model of the HIV-2 integrase (pdb: 3F9K) was modified using the ViewerLite software. The residues involved in IN enzymatic activity were highlighted, as well as positions 91 and 175, which were mutated under RAL pressure. The N-terminal and catalytic domains are represented as: A. Line ribbons; B. Sticks; C. Molecular surface.

IN was then modeled three-dimensionally using the ViewerLite software. 3D-modelling revealed that residue 91 is located on the surface of the enzyme, at the entrance of a pocket that hosts the DDE catalytic triad, and that residue 175 lies in a hydrophobic core region (Figure 3). In such a model, a positive charge at residue 91 could compromise the affinity between RAL and the enzymatic pocket and the I175M substitution may emerge to facilitate access of RAL to the enzymatic pocket [33]. It is possible that the genetic context of the ROD strain, or its relative replicative capacity in the presence of RAL, might favor the emergence of strains harboring the Q91R+I175M mutations over other resistance pathways including the Q148 H/R/K and/or N155 H substitutions. Whether the 91R+175M mutations can be selected in other HIV-2 strains, as well as the relative impact of each individual mutation on sensitivity to RAL, to other INIs, and their respective impact on replicative capacity, require further investigation. The potential selective advantage of each pathway within different contexts is currently being investigated.

## Conclusions

In this study, we described HIV-2 IN polymorphisms detected in group A clinical isolates from 39 patients and in group B isolates from 6 patients. Our data strengthen a previous report on HIV-2 IN polymorphisms [14] and highlight the importance of those residues that remain fully conserved across HIV types and subtypes/groups, including the HHCC Zinc-coordination motif, the DDE catalytic triad and the RKK motif, as well as most residues that ensure enzyme multimerization and correct binding to DNA and positioning. Moreover, we report for the first time the selection of two mutations, Q91R and I175M, under RAL selective pressure. Phenotypic assays with the Q91R + I175M ROD double mutant confirmed the role of these mutations in resistance to RAL showing that they account for a 13-fold decrease in susceptibility to RAL. 3D modeling with ViewerLite indicated that residue 91 lies at the entrance of the pocket that hosts the DDE catalytic triad, and that adding a positive charge at position 91 by switching a Gln to Arg might compromise IN-RAL affinity. How and why these mutations are selected in the context of HIV-2 IN and their relative contribution to resistance to RAL with respect to the more classical mutations at residues 143, 148 and 155 will require further investigation. Taken together, data retrieved from this study should help build more robust HIV-2-specific algorithms for the genotypic interpretation of INI resistance.

## Methods

### Patients and sequences

Fourty-six HIV-2-infected, INI-naïve patients from the Belgian, Luxembourg and Malian cohorts were included in this study. For 7 patients from Belgium and Luxembourg, longitudinal samples were available, and the earliest treatment-naïve (baseline) and latest treatment-experienced samples were selected for sequencing (i.e. 53 samples were sequenced). The full *pol *gene from 53 samples was sequenced from frozen plasma collected between 1997 and 2008: 36 IN sequences (from 29 patients) were from the Belgium and Luxembourg cohorts, and 17 were from the Malian cohort. Patients originated from different countries, as summarized in Table 1. Thirty-eight IN sequences were retrieved from treatment-naïve patients (baseline) and 15 from treatment-experienced patients: 6 patients infected with HIV-2 group A had been exposed to 2-4 NRTIs, 8 patients (7 infected with HIV-2 group A and one infected with a group B strain) were treated with 2-4 NRTIs + 1 or 2 PIs, and for one patient infected with HIV-2 group A, treatment was unknown. PR and RT were sequenced as well and PR-RT sequences are available for 48 of the 51 samples (36 PR-RT sequences for treatment-naïve samples and 14 PR-RT for the treatment-experienced samples. Variability and polymorphisms were defined with respect to the ROD (Genbank X05294) and EHO (U27200) reference sequences, representing HIV-2 group A and group B respectively.

Sequence alignment and phylogenetic analyses indicated that the longitudinal sequences from patients for which 2 samples (one baseline and one treatment-exposed) were available clustered together, as expected. However, for one patient, the 2 longitudinal sequences (GU966548 and GU966572) also clustered with the sequence (GU966559) from one treatment-naïve patient with whom no common history was documented (sequences GU966548 and GU966572 differed from sequence GU966559 by 8 and 4 positions respectively). In order to exclude the risk of biases due to potential contamination, sequence GU966559 was excluded from further analyses. Therefore, 52 IN sequences from 45 patients were maintained for this analysis.

### RNA amplification and sequencing

Whole blood was collected in EDTA-tubes, plasma and cell pellets were separated by centrifugation and stored at -80°C until use. 1 ml of plasma was ultracentrifuged for 1 hour at 25,000 g; RNA was extracted and purified using the QIAamp Viral RNA kit (QIAGEN, Hilden, Germany) and eluted in 50 ul of elution buffer. 10 μl were reverse transcribed to amplify the IN or the PR-RT coding regions using Super-Script One-Step RT-PCR with 2.5 U Platinium Taq (Invitrogen Life Technologies, Carlsbad, California) in a 50 μl mix containing 50 pmol of outer primers. The PR-RT region was amplified as described previously [34]. For IN RNA amplification, forward primer JR25 (5'-GCACCTCCAACTAATCCT-3', nucleotide 2528 of the ROD sequence) and reverse primer JR47 (5'-ATTACCCTGCTGCAAGTCCACC-3', ROD nt 5041) were used for the RT-PCR step and 2 μl of cDNA were amplified using 2.5 U Platinium Taq with forward primer H2Mp9 [34] and reverse primer JR46 (5'-ATGCCCATCCCACCTTATGGTG-3', ROD nt 5019). The IN and PR-RT PCR products were purified on a Microcon column (Millipore, Molsheim, France). The following primers were used to sequence the PR and RT coding regions: forward primers H2Mp3, H2Mp6 and H2Mp9, and reverse primers H2Mp4, H2Mp5, H2Mp7, H2Mp8 and H2Mp10 [34]. For the IN regions, forward primers H2Mp9 [34], JR44 (5'-GAGACCTTCTACACAGATGG-3', ROD nt 3689), JR45 (5'-TATGTTGCATGGGTCCCAGC-3', ROD nt 3971) and AV33 (5'-GTGAAGATGGTAGCATGGTGG-3', ROD nt 4433), and reverse primers JR46 (5'-ATGCCCATCCCACCTTATGGTG-3', ROD nt 5019), and JR48 (5'-GTTCTATACCTATCCACC-3', ROD nt 4466) were used. Sequencing reactions were performed using the Big Dye Terminator cycle-sequencing kit 3.1 on an ABI 3130 xl sequencer following the manufacturer recommendations (Applied Biosystems-Life Technologies, Carlsbad, California). The nucleotide sequences were aligned against the HIV-2 ROD and EHO strains, and mutations were searched using the IDNS (Integrated Database Network System) from Smartgene (Zug, Switzerland).

### Genbank accession numbers

The complete IN coding sequences of the 53 sequenced HIV-2 samples are available in Genbank under the accession numbers GU966535 through GU966581 group A and HM771234 through HM771239 for group B; the ROD-Q91R+I175M double mutant Genbank accession number is HM771240. Forty-seven PR-RT sequences are available under Genbank accession numbers between EF611309 to EF611333[12], and from HQ451906 to HQ451937. Additional file 1: 'Correspondence between Genbank IN and PR-RT accession numbers', summarizes the correspondences between PR-RT and IN sequences.

### Subtyping and phylogenetic analyses

The HIV-2 group was determined for the 3 genes PR, RT and IN through clustering analyses using RAxML v. 7.0.4 and the GTRGAMMA model, rapid bootstrapping (100 runs), and maximum likelihood selection of the optimal tree according to the Rega and Star algorithms.

Phylogenetic analyses of the IN sequences were performed as follows: the appropriate substitution model for the phylogenetic tree was selected with TOPALi v. 2.5. The Akaike information criterion (AIC) and the bayesian information criterion (BIC) chose the GTR model with invariant sites and rate variation among sites. The tree was calculated using RAxML v. 7.0.4 with 100 bootstrap replicates and is included as additional file 2: 'Phylogenetic analysis of the HIV-2 group A and group B IN sequences'.

### Statistical analyses

Statistical analyses were performed using R v.2.8.1. The number of variable positions between IN and RT and IN and PR was compared using a Fisher exact test, and p values < 0.05 were considered statistically significant.

Shannon's entropy at each position was calculated using the Los Alamos Database sequence Entropy website http://www.hiv.lanl.gov/content/sequence/ENTROPY/entropy_one.html for group A and group B strains. Because of the small sample size, variable positions and positions tolerating at least 2 AA changes are highlighted. When polymorphisms were found in sequential samples (one treatment-naïve and one treatment-experienced) from the same patient, they were counted only once, in the treatment-naïve group.

### Viral culture under drug-selective pressure

MT-4 cells [35-37] were obtained through the AIDS Research and Reference Reagent Program, Division of AIDS, NIAID, NIH: MT-4 from Douglas Richman. RAL (monopotassium salt) was supplied by Merck & CO, INC (NJ, US).

Twice a week, 2 × 10^6 ^MT-4 cells were infected with 2 × 10^8 ^TCID_50 _of HIV-2 ROD (obtained from the NIH AIDS Research Reagent Program) in 2 ml of RPMI-1640 medium supplemented with 2 mM glutamine and 50 ug/ml gentamicin, 10% fetal clone 1 bovine serum (all from Gibco - Invitrogen, Paisley, UK). After at least two hours of incubation at 37°C in 5% CO_2 _(gently mixed every hour), cells were washed with PBS and resuspended in 10 ml of culture medium in the presence of RAL. The initial RAL concentration (0.001 nM) was lower than the mean ROD IC_50 _(0.00395 nM). Virus titers were determined using a previously described real-time PCR protocol [38,39]. Drug concentrations were raised gradually by 3-fold and at each drug increment, two separate cultures were maintained, one with the former concentration of drug (back-up culture) and one with the new, increased concentration. If the viral titer remained stable during 5 successive passages at the higher concentration, the drug level was increased further by 3-fold. Sequencing of the IN coding region was performed as described above, using 1 ml of culture supernatant.

### Phenotypic sensitivity to RAL

#### MTT assay

RAL inhibitory effect (IC_50_) on the HIV-induced cytopathic effect was assessed in MT-4 cell cultures using an MTT assay as previously described [40]. The assay is based on the reduction of the yellow MTT (3-[4,5-dimethylthiazol-2-yl]-2,5-diphenyl tetrazolium bromide) to purple formazan by living cells. The parental HIV-2 ROD was used as a reference. 30 × 10^4 ^MT-4 cells/well of a 96-well flat-bottomed plate were infected in quadruplicate wells with 50 ul of viral culture supernatant, in the presence of 0.075% sodium bicarbonate and 1% hepes (both from Gibco - Invitrogen, Paisley, UK) and of serial 3-fold dilutions of RAL ranging from 3.763 × 10^-5 ^nM to 20 nM. After 3 days of incubation at 37°C in 5% CO_2_, 150 l of supernatant were removed carefully from each well without disturbing the cells. Thirty l of MTT solution were added to each well (*in vitro *cytotoxicity assay kit MTT based, Sigma-Aldrich, St-Louis, MO, USA) and the plate was incubated for 4 hours at 37°C in 5% CO_2_. Formazan crystals were then solubilized with 100 ul of acidified isopropanol (HCl 0.1 N, Triton X-100 10% V/V) and by shaking the plate during 10 minutes. Absorbance was measured at 540/_690 _nm and the percentage of protection (PP = (O.D. measured - O.D. infected cells without RAL)/O.D. uninfected cells - O.D. infected cells without RAL) × 100). Mock infected cells are expected to strongly reduce the MTT substrate and to produce the highest O.D., reflecting the highest cell survival level in the absence of virus; infected cells in the absence of RAL, in contrast, are unprotected and thus minimally reduce the MTT substrate, as reflected by O.D. measures comparable to background. With increasing RAL levels, an increasing number of cells is expected to be protected from HIV-induced cytopathic effects, and the O.D. is expected to increase. RAL IC_50 _was computed using the GraphPad Prism 5 software (GraphPad Software, San Diego, California, USA).

### 3-D modeling

A 3D-model of the HIV-2 integrase (pdb: 3F9K) was modified using the ViewerLite software.

### Ethical approval

The present study was conceived according to the Helsinki Convention norms and was approved by the ethical committee of the Faculté de Médecine, de Pharmacie et d'Odontostomatologie of the University of Bamako (Mali), and by the biomedical ethical commission of the UCLouvain (Brussels, Belgium) - 2009/04MAR/084 B40320096021.

## Competing interests

The authors declare that they have no competing interests.

## Authors' contributions

DPB analyzed the data and wrote the publication. PT performed the *in vitro *phenotypic assays and contributed to the drafting. CL performed the sequencing. AAO took the samples and collected the data in Mali. AMT performed the statistical analyses. SD supervised the project in Mali. PG followed patients in Belgium, contributed patient samples and clinical data, and reviewed the manuscript. JCS followed patients in Luxembourg, contributed patient samples and clinical data, reviewed the manuscript. JR supervised the project, analyzed the data and contributed to the drafting.

## Supplementary Material

Additional file 1 **Correspondence between Genbank IN and PR-RT accession numbers**. Genbank IN and PR-RT accession numbers, as well as some clinical data (including treatment experience and eventually treatment, and country) are reported. Click here for file

Additional file 2 **Phylogenetic analysis of the HIV-2 group A and group B IN sequences**. Phylogenetic analyses of the 53 IN sequences from the 46 HIV-2 infected, INI-naïve patients (Genbank accession numbers GU966535 through GU966581 for group A and HM771234 through HM771239 for group B) were performed using TOPALi v. 2.5. The Akaike information criterion (AIC) and the Bayesian information criterion (BIC) chose the GTR model with invariant sites and rate variation among sites. The tree was calculated using RAxML v. 7.0.4 with 100 bootstrap replicates. The strain SIV MAC.US.x.239.M33262 served as the outgroup. Click here for file
