# Supplementary material for: Polymorphisms of HIV-2 integrase and selection of resistance to raltegravir
Source: Retrovirology. 2010 Nov 29;7:98. doi: 10.1186/1742-4690-7-98 (PMC3006360; doi:10.1186/1742-4690-7-98)
Supplement: Additional file 1 — Correspondence between Genbank IN and PR-RT accession numbers. Genbank IN and PR-RT accession numbers, as well as some clinical data (including treatment experience and eventually treatment, and country) are reported. [file 1742-4690-7-98-S1.DOC]

**Additional file 1: Correspondence between Genbank IN and PR-RT accession numbers.**

| **Access no. INTEGRASE** | **Access no.**  **PR-RT** | **ARV Therapy** | **HIV-2 group** | **Country of sampling** | **Follow-up sample of** |
| --- | --- | --- | --- | --- | --- |
| GU966535 | HQ451906 | ARV Naive | A | Mali |  |
| GU966536 | HQ451907 | ARV Naive | A | Mali |  |
| GU966537 | HQ451908 | ARV Naive | A | Mali |  |
| GU966538 | HQ451909 | ARV Naive | A | Mali |  |
| GU966539 | HQ451910 | ARV Naive | A | Mali |  |
| GU966540 | HQ451911 | ARV Naive | A | Mali |  |
| GU966541 | HQ451912 | ARV Naive | A | Mali |  |
| GU966542 | No seq available | ARV Naive | A | Mali |  |
| GU966543 | HQ451913 | ARV Naive | A | Mali |  |
| GU966544 | HQ451914 | ARV Naive | A | Mali |  |
| GU966545 | No seq available | ARV Naive | A | Mali |  |
| GU966546 | No seq available | ARV Naive | A | Mali |  |
| GU966547 | HQ451915 | ARV Naive | A | Luxembourg |  |
| GU966548 | EF611321 | ARV Naive | A | Luxembourg |  |
| GU966549 | EF611324 | ARV Naive | A | Luxembourg |  |
| GU966550 | EF611320 | ARV Naive | A | Luxembourg |  |
| GU966551 | EF611328 | ARV Naive | A | Luxembourg |  |
| GU966552 | EF611332 | ARV Naive | A | Luxembourg |  |
| GU966553 | EF611322 | ARV Naive | A | Luxembourg |  |
| GU966554 | EF611323 | ARV Naive | A | Luxembourg |  |
| GU966555 | EF611325 | ARV Naive | A | Luxembourg |  |
| GU966556 | EF611326 | ARV Naive | A | Luxembourg |  |
| GU966557 | EF611329 | ARV Naive | A | Luxembourg |  |
| GU966558 | HQ451916 | ARV Naive | A | Luxembourg |  |
| GU966560 | HQ451917 | ARV Naive | A | Luxembourg |  |
| GU966561 | EF611310 | ARV Naive | A | Belgium |  |
| GU966562 | EF611311 | ARV Naive | A | Belgium |  |
| GU966563 | HQ451918 | ARV Naive | A | Belgium |  |
| GU966564 | HQ451919 | ARV Naive | A | Belgium |  |
| GU966565 | HQ451920 | ARV Naive | A | Belgium |  |
| GU966567 | HQ451921 | ARV Naive | A | Belgium |  |
| GU966568 | HQ451922 | ARV Naive | A | Belgium |  |
| HM771238 | HQ451935 | ARV Naive | B | Mali |  |
| HM771237 | HQ451936 | ARV Naive | B | Mali |  |
| HM771239 | EF611312 | ARV Naive | B | Belgium |  |
| HM771235 | No seq available | ARV Naive | B | Luxembourg |  |
| HM771234 | EF611309 | ARV Naive | B | Belgium |  |
| GU966569 | HQ451923 | d4T-3TC-IDV/r | A | Mali |  |
| GU966570 | HQ451924 | AZT-3TC-ABC | A | Mali |  |
| GU966571 | No seq available | AZT-3TC | A | Luxembourg | GU966549 |
| GU966572 | HQ451925 | AZT-3TC-ABC-TDF-DRV/r | A | Luxembourg | GU966548 |
| GU966573 | HQ451926 | AZT-3TC-ABC | A | Luxembourg | GU966558 |
| GU966574 | HQ451927 | 3TC-TDF-LPV/r | A | Luxembourg | GU966552 |
| GU966575 | HQ451928 | AZT-3TC-ABC-SQV/r | A | Luxembourg | GU966550 |
| GU966576 | HQ451929 | AZT-3TC-ABC-FTC | A | Luxembourg | GU966554 |
| GU966577 | HQ451930 | d4T-ABC-LPV/r | A | Belgium | GU966561 |
| GU966578 | EF611333 | d4T-3TC-ABC | A | Belgium |  |
| GU966579 | HQ451931 | d4T-3TC-NFV | A | Belgium |  |
| GU966580 | HQ451932 | 3TC-ABC-TDF | A | Belgium |  |
| GU966581 | HQ451933 | 3TC-TDF-SQV-ATV/r | A | Belgium |  |
| GU966566 | HQ451934 | Unknown treatment | A | Belgium |  |
| HM771236 | HQ451937 | AZT-3TC-LPV/r | B | Mali |  |
