# Supplementary figures and images for: Polymorphisms of HIV-2 integrase and selection of resistance to raltegravir
Source: Retrovirology. 2010 Nov 29;7:98. doi: 10.1186/1742-4690-7-98 (PMC3006360; doi:10.1186/1742-4690-7-98)

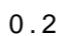

Supplement: Additional file 2 — Phylogenetic analysis of the HIV-2 group A and group B IN sequences. Phylogenetic analyses of the 53 IN sequences from the 46 HIV-2 infected, INI-naïve patients (Genbank accession numbers GU966535 through GU966581 for group A and HM771234 through HM771239 for group B) were performed using TOPALi v. 2.5. The Akaike information criterion (AIC) and the Bayesian information criterion (BIC) chose the GTR model with invariant sites and rate variation among sites. The tree was calculated using RAxML v. 7.0.4 with 100 bootstrap replicates. The strain SIV MAC.US.x.239.M33262 served as the outgroup. [file 1742-4690-7-98-S2.PDF]
